# Incidence and predictors of opportunistic infections in adolescents and adults after the initiation of antiretroviral therapy: A 10-year retrospective cohort study in Ethiopia

**DOI:** 10.3389/fpubh.2022.1064859

**Published:** 2022-12-15

**Authors:** Beshada Zerfu Woldegeorgis, Chala Wegi Diro, Bereket Yohannes, Amene Abebe Kerbo, Yordanos Sisay Asgedom

**Affiliations:** ^1^School of Medicine, College of Health Sciences and Medicine, Wolaita Sodo University, Sodo, Ethiopia; ^2^Department of Epidemiology and Biostatistics, College of Health Sciences and Medicine, Wolaita Sodo University, Sodo, Ethiopia; ^3^School of Public Health, College of Health Sciences and Medicine, Wolaita Sodo University, Sodo, Ethiopia

**Keywords:** opportunistic infections, Ethiopia, incidence, antiretroviral therapy, HIV/AIDS

## Abstract

**Background:**

Opportunistic infections (OIs) are the leading cause of morbidity and mortality in people living with the human immunodeficiency virus (PLHIV). However, there are few robust recent data on the rates of OIs and the risk factors that contribute to their occurrence. Therefore, the current study sought to determine the incidence of OIs and identify predictors among adolescents and adults after the initiation of antiretroviral therapy (ART) at Wolaita Sodo University Comprehensive Specialized Hospital (WSUCSH), Southern Ethiopia.

**Methods:**

A retrospective cohort study design was employed. The study population was adolescents and adults who initiated ART between 1 January 2012 and 31 December 2021. A simple random sampling technique was used to select 537 participants' records. We reviewed the medical records of the sampled individuals from 1 May 2022 to 15 June 2022. KoboCollect version 2021.2.4 and STATA version 14.0 software were used for data collection and analysis, respectively. We calculated the incidence rate per 100 person-years of observation (PYO) with 95% confidence intervals (CIs) for the occurrence of any OIs. The Weibull regression model was fitted after the goodness-of-fit test for the Cox proportional hazard model was deemed inadequate. An adjusted hazard ratio (AHR) with 95% CI was used to identify a significant predictor of OIs. The statistical significance was made at a 5% significance level.

**Results:**

A total of 515 participants contributed to 1,829 person-years of risk, of whom 164 (31.84%) exhibited at least one OI. The overall incidence rate of OIs was 8.97 cases (95% CI: 7.69, 10.44) per 100 PYO. The independent predictors of OIs were being female [AHR: 1.65 (95% CI (1.15, 2.36), *P* = 0.007)], individuals classified as World Health Organization (WHO) HIV clinical stage III [AHR: 1.98 (95% CI (1.12, 3.51), *P* = 0.019)], individuals who did not take cotrimoxazole preventive therapy (CPT) [AHR: 2.58 (95% CI (1.62, 4.11), *P* < 0.001)], mild malnutrition [AHR: 1.62 (95% CI (1.06, 2.54), *P* = 0.035)], and poor adherence to ART [AHR: 4.21 (95% CI (2.39, 7.44), *P* < 0.001)].

**Conclusion:**

The rate of OIs after the initiation of ART was still high. Moreover, being female, not taking CPT, poor adherence to ART, mild malnutrition, and advanced HIV disease at presentation were found to increase the hazards of developing OIs.

## Background

Human immunodeficiency virus (HIV) causes progressive immune cell depletion and reduces a person's ability to combat opportunistic infections (OIs) (1). Over 20 specific OIs have been linked to HIV (2), and people living with human immunodeficiency virus (PLHIV) are frequently exposed to co-infections during the disease (3). Moreover, HIV-related OIs affect PLHIV more frequently and severely, causing significant morbidity and mortality, and necessitating lifelong antiretroviral therapy (ART) (4, 5).

Recent global estimates suggest that 79.3 million people have become infected with HIV since the first evidence of the epidemic. In addition, 36.3 million people have died from acquired immune deficiency syndrome (AIDS)-related illnesses (6). High levels of early morbidity and mortality following ART initiation continue to be a distinctive feature of ART programs in Sub-Saharan Africa, although the introduction and scaling-up of ART have decreased overall mortality (7, 8). The World Health Organization (WHO) recommends a range of medical interventions to reduce the occurrence of OIs among PLHIV. These include, but are not limited to the reduction of exposure, chemoprophylaxis, immunization, and rapid initiation of ART (9).

In concert with these, Ethiopia has adopted several strategies to improve the HIV care continuum, including (a) launching ART services in 2003, (b) declaring universal access to free ART in 2005 which is reflected in part in the increased ART coverage from 5% in 2010 to 9.5% in 2013, (c) treating all policies since 2016 to improve the survival of HIV, (d) endorsement of the national HIV prevention 2020 road maps, and (e) rollout of dolutegravir (DTG) and third-line ART regimen; optimization of pediatric and adult ART, and phase out of nevirapine (NVP) (1, 10). OIs are the leading cause of hospitalization and death in patients with HIV and still present formidable challenges for meager healthcare systems endeavoring to provide effective and efficient HIV care (11, 12).

In resource-poor settings, HIV-related OIs occupy between 20 and 52% of hospital beds (13). Furthermore, 90% of HIV-related morbidity and mortality are attributed to OIs (14, 15). Between 2000 and 2019, US $170.79 billion in development assistance for health was spent on HIV globally and most of this aid went toward care and treatment for patients with HIV (16). Moreover, OIs have significantly contributed to poverty among PLHIV, impeding the achievement of sustainable development goal III on health in resource-poor settings (17). Since the advent of ART, a drastic decrease in rates of OIs was observed, but still, the incidence rate of OIs in industrialized countries differs markedly from those countries in Africa (18). In Poland, for instance, the rate of OIs was 6.8 in 2000, 6.5 in 2001, and 4.8 in 2002 per 100 person-years of observation (PYO) (19). Furthermore, the rate of OIs per 100 PYO was 4.67 in Vietnam (20); 2.27 in the US and Canada (2000–2010) (21); 6.9 in London (22); and 7.7 at 3 months, 2.6 at 6 months, and 2.2 at between 9 and 15 months in Swiss HIV cohort study (23).

A few studies demonstrated that the OIs in low and middle-income countries remain the major driver of HIV-associated morbidity and mortality (24). In Uganda, the rate of OIs was 5.9 cases per 100 PYO (25). In the Senegalese cohort, the rates of AIDS-defining illness decreased from 20.5 to 4.3 per 100 PYO between the first and fourth year (26). According to previous studies, but limited studies conducted in Ethiopia, the incidence of OIs was 13.5 cases per 100 PYO in Dessie, Northern Ethiopia (27), and 3.4 cases per 100 PYO in Arba Minch, Southern Ethiopia (28). Although ART has been proven to be impactful in halting immune system impairment and preventing disease progression, studies showed that OIs have not ended (1, 29) either due to the unmasking of subclinical infection that occurs with immune recovery, drug toxicities and interactions, initial acquisition of a drug-resistant strain, or high exposure to infectious agents (10, 30).

A study in the United Kingdom (UK) reported that late presentation to health facilities increases the risk of developing OIs and remains a significant problem in developed countries, with over 20% of patients in the UK suffering from OIs (31). In addition, baseline NVP-based regimens, time-varying higher viral load, treatment failure, and time-varying lower hemoglobin levels were risk factors associated with the development of OIs at any given time, while the patient is taking ART (25, 32, 33).

Furthermore, adherence to ART, nutritional status, isoniazid preventive therapy (IPT) and cotrimoxazole preventive therapy (CPT), place of residence, functional status, gender, age of the PLHIV, CD4 T lymphocytes count, and disclosure status have been associated with the occurrence of OIs among adolescent and adult PLHIV after the initiation of ART (27, 28, 32, 34). Reliable data on the burden of OIs after ART initiation are critical for planning health services and reducing OI-related morbidity and mortality. Nevertheless, information on the rates of OIs is scarce nationally and non-existent in the study setting.

In this study, we sought to determine the incidence of OIs and identify potential risk factors correlated with the acquisition of OIs among adolescent and adult participants in HIV care. A follow-up study, like the current study, would make difference to healthcare professionals, who are caring for patients infected with HIV receiving ART at Wolaita Sodo University Comprehensive Specialized Hospital (WSUCSH), in two regards. In the first instance, to predict potential risk factors for the acquisition OIs. Second, it enables healthcare professionals to remain knowledgeable about the estimated time of OI occurrence after ART initiations which in turn helps design the optimal strategies for the prevention and management of OIs and provide comprehensive and high-quality care to patients with HIV. Furthermore, the results obtained from this study will enable administration bodies to define local priorities in HIV care and inform targeted expenditures on the prophylaxis and treatment of HIV-related OIs. In addition, evidence is expected to be used by policymakers and program managers mainstreaming HIV/AIDS activities. Finally, the current study calls for researchers to conduct a multi-centered prospective follow-up study.

## Methods

### Study design

An institution-based retrospective cohort study design was employed.

### Study setting

The study was conducted at WSUCSH, which is located in Wolaita Zone, Sodo town, 325 km south of Ethiopia's capital, Addis Ababa. A total of 28 public health facilities, including one specialized hospital, were available in the town. The hospital provides inpatient and outpatient services for an estimated five million people, including patients visiting from neighboring zones and regions of Ethiopia. Comprehensive HIV care and treatment were launched in September 2005 at the hospital. A total of 26 staff, comprising four ART physicians, four ART nurses, two data clerks, two porters, two cleaners, two case managers, four ART adherence supporters, two ART pharmacists, two clinical officers, and two ART laboratory technologists, were providing healthcare services at the hospital's ART clinic. A total of 1,897 (male participants-−828; female participants-−1,069) PLHIV were initiated on ART from 1 January 2012 to 31 December 2021, at the center. Of these, 60 individuals were younger than 15 years. We reviewed the medical records of the sampled individuals from 1 May 2022 to 15 June 2022. This study was reported following the Strengthening the Reporting of Observational Studies in Epidemiology (STROBE) statement (available online at https://www.strobe-statement.org/).

### Participants

Eligible PLHIV were individuals whose age was at least 15 years old (adolescents and adults) and who initiated ART at WSUCSH between 1 January 2012 and 31 December 2021. Records of PLHIV with missing information, such as an unknown ART start date, OIs that were not documented, or an incomplete diagnosis, were excluded from data extraction.

### Sample size and sampling procedure

**Figure 1:**
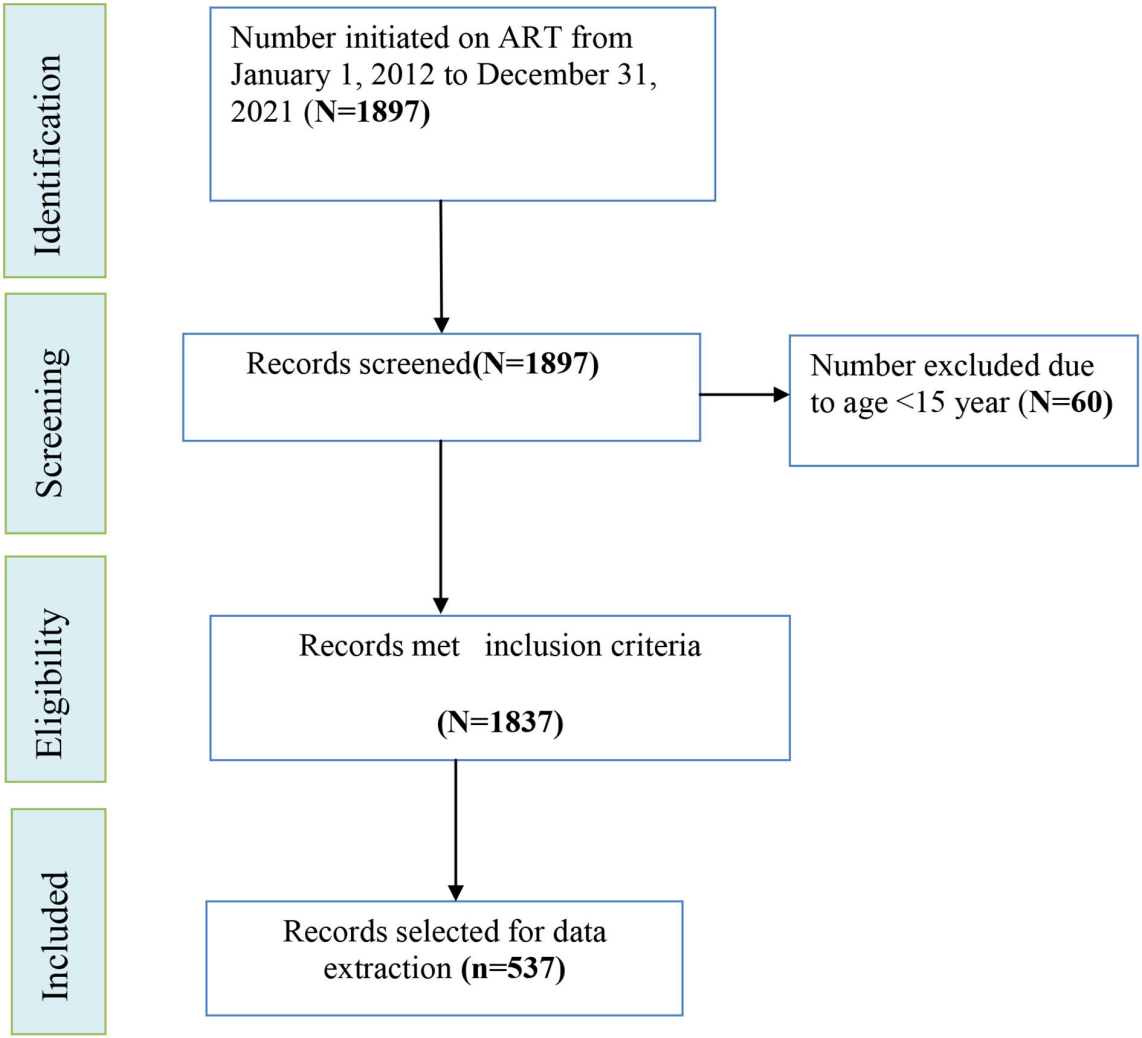
Schematic illustration of sampling procedure for incidence and predictors of OIs among adolescents and adults after the initiation of ART at WSUCSH, Southern Ethiopia, 2012–2021.

We computed the required sample size directly using OpenEpi version 3.0.1 (Open Source Epidemiologic Statistics for Public Health) software designed for cohort studies based on the following assumptions: The frequency of outcome in the unexposed was 50%; the frequency of outcome in the exposed was 63%; absolute precision of 5%; Zα/2 = 1.96 at 95% confidence interval (CI), and a power of 80%. The variable that resulted in a larger sample size from the previous study conducted in Ethiopia was the body mass index (BMI) of the participants (35). Considering a 10% contingency for incomplete information, the final sample size for this study turned out to be 537. Between 1 January 2012 and 31 December 2021, a total of 1,897 patients initiated ART. Of which, records of 60 PLHIV under the age of 15 years were excluded (Figure 1). Medical records of 537 participants were selected from the remaining 1,837 using the simple random sampling method. To prepare the sampling frame, sequential numerical codes starting with 1 and ending with 1,837 were assigned to the eligible charts found on the shelves. Microsoft Excel 2010 was used to create random sampling. When the “RAND function ()” was entered, random numbers were created. After sorting them, the required 537 samples of random numbers were selected for data extraction.

### Study variables

The outcome variable of this study was time to the acquisition of OIs. The time was estimated in years, and it was from the date when the patient was initiated on ART to the date when OI was diagnosed. Opportunistic infections are infections that occur more frequently and severely among PLHIV with weakened immune systems (5). Participants with any of the following infections, herpes zoster, candidiasis (oral or vaginal), cryptococcal meningitis, chronic diarrhea, encephalopathy, herpes simplex, PCP, pneumonia, pulmonary tuberculosis, extrapulmonary tuberculosis, intestinal parasitosis, acute diarrhea, toxoplasmosis, and upper respiratory tract infection, were considered as having OIs (event) only if diagnosed and documented by ART physician (10) and therefore coded as “1,” whereas those who dropped, lost to follow-up, transferred out, died, or were still on active ART follow-up but did not develop any OIs until the end of the follow-up were considered censored and coded as “0.” The information on the outcome variable was obtained by reviewing the patient chart documented by the ART physician. The explanatory variables were categorized into the following themes: socio-demographic variables: age, gender, marital status, level of education, residence, religion, occupation, and catchment area; clinical, treatment, and laboratory-related variables: WHO HIV clinical stage, BMI, baseline hemoglobin, ART adherence, functional status, IPT, CPT, type of first-line ARV regimen, prior history of OIs, and disclosure status.

### Measurements

An electronic data collection system using KoBoCollect version 2021.2.4 software was employed to retrospectively abstract data on exposure and outcome variables that had been filled in properly by medical staff at enrollment and each visit of the patients. Published studies (27, 28, 32), patient cards, and the national HIV care/ART intake and follow-up forms, which are available in the ART clinic, were sources of information to create the XLSForm Online. The XLSForm uploaded to the server contains socio-demographic variables such as age, sex, residence, educational background, marital status, and occupation; clinical and laboratory-related variables such as weight, height, WHO HIV clinical stage, baseline hemoglobin, comorbidity, ART adherence, and functional status. Treatment-related variables such as IPT, CPT, and baseline ART regimen were all factors to be considered. The quality of the data was ensured by using pre-tested and refined checklists. Before the actual data collection, a pre-test was conducted to prove its internal validity.

The levels of reliability agreements among the data collectors were analyzed using statistical packages for the social sciences (SPSS) version 25 after retrieving data on a selected variable from 5% (*n* = 26) of the patient records and were verified by using Cohan's kappa coefficient. The standard for the strength of agreement for the kappa coefficient was categorized as poor ( ≤ 0.20), fair (0.21–0.40), moderate (0.41–0.60), good (0.61–0.80), and almost perfect agreement (0.81–1). A kappa statistic value of ≥0.5 was considered congruent and accepted (64).

The reliability analysis revealed that there was a statistically significant similarity between the two data extractors (Cohen's kappa = 87.4%, *P* < 0.001). Data collectors and supervisors were trained intensively for 1 day which emphasized the relevance of the study, the objective of the study, how to use KoBoCollect software, and the overall data extraction procedure. Meetings between supervisors and data collectors were held to address preemptive problems. The overall data collection process was monitored by the principal investigator and the supervisors.

### Definition of terms

#### Censored

Censored was recorded if patients were dropped, lost to follow-up, transferred out, died, or were still on active ART follow-up but did not develop any OIs until the end of the follow-up (36). Transferred out was recorded when patients with HIV-positive on follow-up care in one health institution were transferred to another to continue their follow-up there; loss to follow-up occurred when PLHIV on HIV care was not seen for at least 1 month, and drop-out occurred when PLHIV on HIV care was not seen for at least 3 months as recorded by ART clinic personnel (10).

#### Event

Event, also usually known as a failure, was entertained if patients infected with HIV on ART developed any form of OI during the follow-up period (37).

#### Time

Time, also known as survival time, was the time in years/months/days between the start of ART for PLHIV and the occurrence of the outcome. It refers to the time variable as survival time, and it indicates how long a patient infected with HIV on ART “survived” during the follow-up period (37).

#### ART adherence

It was classified as good, fair, or poor, according to the percentage of drug dosage calculated from the total monthly dose of ART drugs. Good ART adherence was reported with compliance ≥95% or ≤ 3 missed doses per month; fair adherence to ART reflects 85–94% compliance or between 4 and 8 missing doses per month, and poor adherence reflects < 85% compliance or ≥ 9 missed dose per month (10).

#### Functional status

It was classified as “working” if the individual was able to perform usual work in or out of the house; “ambulatory” can perform activities for daily living, and “bedridden” if unable to perform activities of daily living (10).

#### WHO HIV clinical staging

It is the way to categorize HIV disease severity based on new or recurrent clinical events. There are four WHO clinical stages ranging from asymptomatic (WHO stage I) to severe symptoms (WHO clinical stage IV) (9).

#### Body mass index (BMI)

Body mass index was computed as per WHO adopted national ART guideline, that is, weight (kg)/height (m)^2^. Thus, patients were grouped based on their baseline BMI into six categories: 18.5–24.99 (normal), 17–18.49 (mild malnutrition), 16–16.99 (moderate malnutrition), < 16 (severe malnutrition), 25–29.99 (overweight), and ≥30 (obese) (10). Anemia is defined as having a hemoglobin level of < 10 gm/dl (38).

### Statistical methods

Data collected and entered simultaneously *via* electronic systems were downloaded as an XLS and exported to STATA Version 14.0 (Stata Corp. LP, College Station, Texas) for statistical analysis. Before analysis, the data were arranged, edited, and cleaned by running simple frequencies and cross-tabulations. Furthermore, distributional plots and tests and categorization for quantitative variables (age, baseline weight, and hemoglobin) were performed; data were declared to be survival-time data. The incidence rate of OIs was calculated by dividing the number of events experienced by the person-years at risk.

The overall Kaplan–Meier (KM) survival curves (product limit estimate) with 95% CI, stratified by categorical covariates, were plotted to demonstrate how the survival experience varied, and the equivalence of KM survival curves was tested using the generalized log-rank (Mantel–Cox). Mathematical assumptions associated with the cox proportional hazards model were explicitly validated which include the independence of observation and proportional hazards. Thus, there were no multicollinearity problems (mean VIF = 1.71) attesting that observations were independent.

The proportional hazards assumptions for each variable and globally for all predictors were tested using the Schoenfeld residuals. Thus, when fitted, the global test was insignificant (*P* = 0.0001), indicating a violation of the assumption that hazards are proportional (assumptions not satisfied). This model finding was further confirmed by the Cox–Snell Residual Nelson–Aalen Cumulative hazard graph. The proportional hazard assumptions were also graphically evaluated for different categories of variables being investigated using log–log survival curves, comparison of “observed” with “expected” survival curves, and time-dependent covariates, all of which failed to satisfy this assumption.

The lowest value of Akaike's information criterion and Bayesian information criterion was used to determine which best fit model from the parametric distributions is selected. Accordingly, the Weibull regression model was fitted. Before multivariable regression analyses, the data were fitted to check the adequacy of the Weibull model by stratified KM curves.

The Weibull regression diagnostic plot revealed that the lines for stratified covariates are generally parallel and linear in their scale attesting that the model was fit. In the bi-variable Weibull regression analysis, variables with *p* ≤ 0.25 were selected and fitted in the multivariable Weibull regression model to control for possible confounding effects.

In the final model, variables with *p* < 0.05 were considered statistically significant predictors of OIs. The effect of each variable was estimated using adjusted hazard ratios (AHRs) with a 95% CI.

## Results

### Characteristics of the study participants

We analyzed data extracted from the charts of 515 (95.9%) HIV-infected adolescents and adults who initiated ART between 1 January 2012 and 31 December 2021. Among the censored individuals, 276 (78.63%) were as a result of the end of the follow-up period; 40 (11.43%) were due to transfer to another health facility; 21 (5.99%) were as a result of dropping out; and 13 (3.7%) were due to being lost to follow-up. The majority of those studied, 295 (57.3%), were female subjects. The median age at baseline was 30 years [interquartile range (IQR), 27–38 years], 311 (60.4%) were married, 106 (20.6%) were merchants, and 432 (83.9%) were living within the catchment area (Table 1).

**Table 1:** Baseline socio-demographic characteristics of participants at the initiation of ART at WSUCSH, Southern Ethiopia, 2012–2021 (*n* = 515).

**Characteristics**	**Category**	**Frequency**	**Percentage**
Age (years)	15–24	63	12.2
	25–34	246	47.8
	35–44	150	29.1
	45–54	43	8.3
	≥55	13	2.5
Gender	Female	295	57.3
	Male	220	42.7
Marital status	Married	311	60.4
	Never married	93	18.1
	Divorced	68	13.2
	Widowed	34	6.6
	Separated	9	1.7
Educational level	No education	106	20.6
	Primary	201	39.0
	Secondary	126	24.5
	Tertiary and above	82	12.9
Occupation	Housewife	139	27.0
	Merchant	106	20.6
	Government employee	87	16.9
	Daily laborer	57	11.1
	Farmer	49	9.5
	No work	50	9.7
	Other	26	5.0
Residence	Urban	406	78.8
	Rural	109	21.2
Living within the catchment area	Yes	432	83.9
	No	83	16.1

The baseline clinical, laboratory, and treatment-related characteristics of the participants are presented below. A total of 187 (36.3%) patients were enrolled in chronic ART after the test and treatment WHO recommendation. Isoniazid preventive therapy and CPT were initiated in 334 (64.85%) and 466 (90.49%) patients, respectively. The median body weight at baseline was 56 kg (IQR, 48–63 kg), 413 (80 %) were able to perform their usual work, 220 (44%) were in WHO HIV clinical stage I at presentation, and 330 (64.1%) were started on tenofovir (TDF) + lamivudine (3TC) + efavirenz (EFV) regimen (Table 2).

**Table 2:** Clinical, laboratory, and treatment-related characteristics of participants on ART at WSUCSH, Southern Ethiopia, 2012–2021 (*n* = 515).

**Characteristics**	**Category**	**Frequency**	**Percentages**
Ever initiated on CPT	Yes	466	90.5
	No	49	9.5
Ever initiated on IPT	Yes	334	64.9
	No	181	35.2
Hemoglobin	< 10 gm/dl	126	24.5
	≥10 gm /dl	389	75.5
Nutritional status	Normal	306	59.4
	Mild malnutrition	80	15.5
	Moderate malnutrition	33	6.4
	Severe malnutrition	46	8.9
	Overweight	42	8.2
	Obese	8	1.6
Functional status	Working	413	80.2
	Ambulatory	92	17.9
	Bedridden	10	1.9
WHO HIV clinical stage	Stage I	226	43.9
	Stage II	109	21.2
	Stage III	152	29.5
	Stage IV	28	5.4
ART adherence	Good	450	87.4
	Fair	39	7.6
	Poor	26	5.1
Baseline ARV regimen	AZT + 3TC + NVP	29	5.6
	AZT + 3TC + EFV	15	2.9
	TDF + 3TC + EFV	330	64.1
	TDF + 3TC + NVP	21	4.1
	TDF + 3TC + DTG	120	23.3
Comorbidity	No	500	97.1
	Yes	15	2.9
Presence of OI at enrollment	Yes	303	58.8
	No	212	41.2

### Person-times at risk for the development of OIs

Participants were followed until they were censored or experienced an event after the initiation of ART was identified. The median duration of follow-up time was 36.9 months (IQR, 12.5–69.6 months). During 10 years (2012–2021), 515 persons contributed 1,829 PYO or 22,252.833 person-months at risk and 164 (31.84%) exhibited at least one OI.

### Incidence and type of opportunistic infections

**Figure 2:**
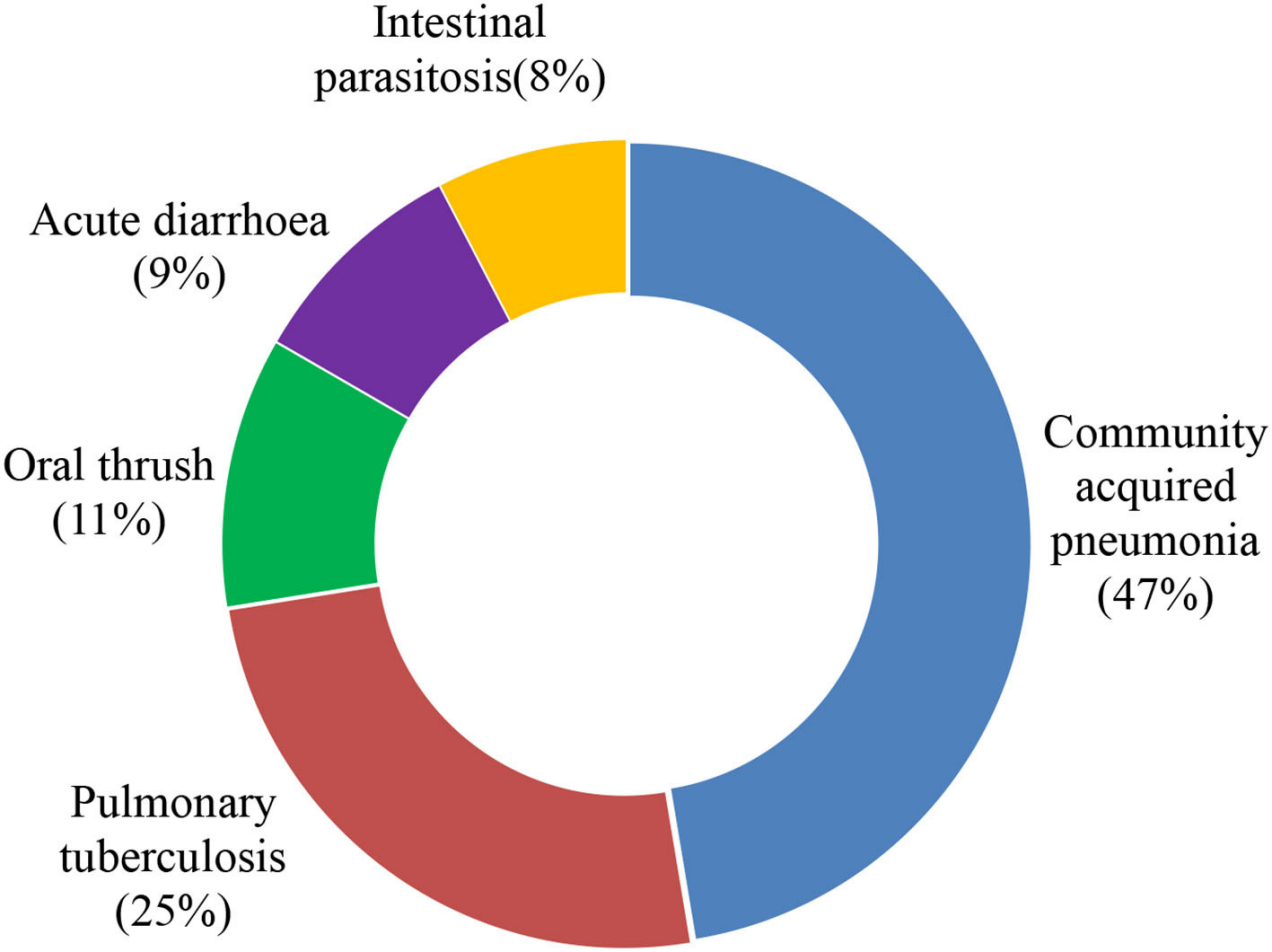
Common type of OIs identified among adolescents and adults after the initiation of ART at WSUCSH, Southern Ethiopia, 2012–2021.

The overall person-time incidence rate of OIs among adolescents and adults after the initiation of ART was found to be 8.97% (95% CI: 7.69, 10.44) per 100 PYO. Community-acquired pneumonia was the predominant, 68 (41.5%) OIs assessed during the follow-up period, followed by pulmonary tuberculosis, 36 (22%), and oral thrush, 16 (9.5%) (Figure 2). Furthermore, chronic diarrhea (6.1%), herpes zoster (5.5%), esophageal candidiasis (4.9%), cryptococcal meningitis (3.7%), and tinea capitis (0.6%) were also retrieved from the records. In the majority of the PLHIV, 140 (85.4%) developed a single OI, while 15 (9%) and 10 (6%) developed two and at least three OIs.

### Comparison of opportunistic infection-free survival time

**Figure 3:**
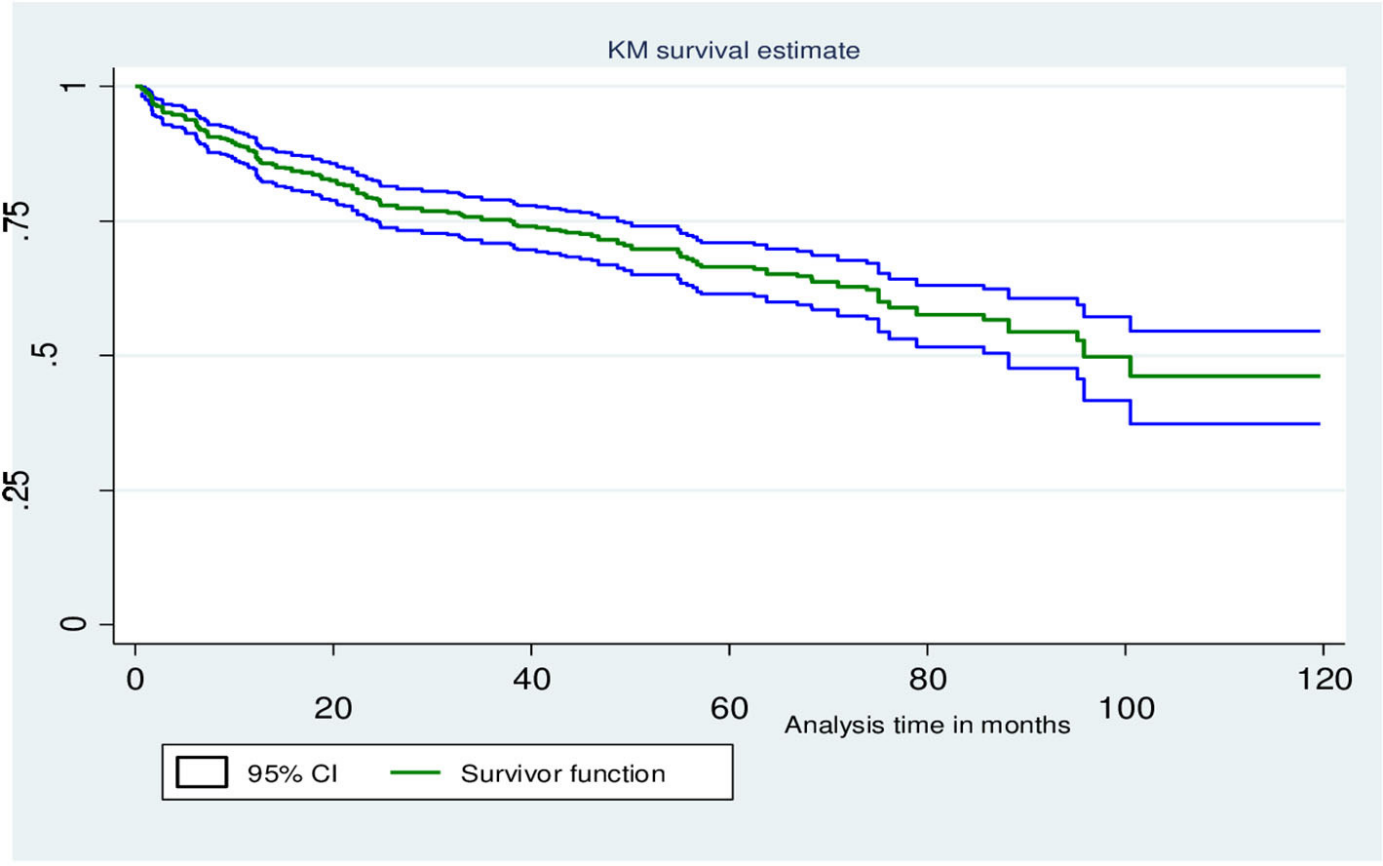
KM survival curves illustrate the probability of OI-free survival over follow-up time for the cohort after the initiation of ART at WSUCSH, 2012–2021.

**Figure 4:**
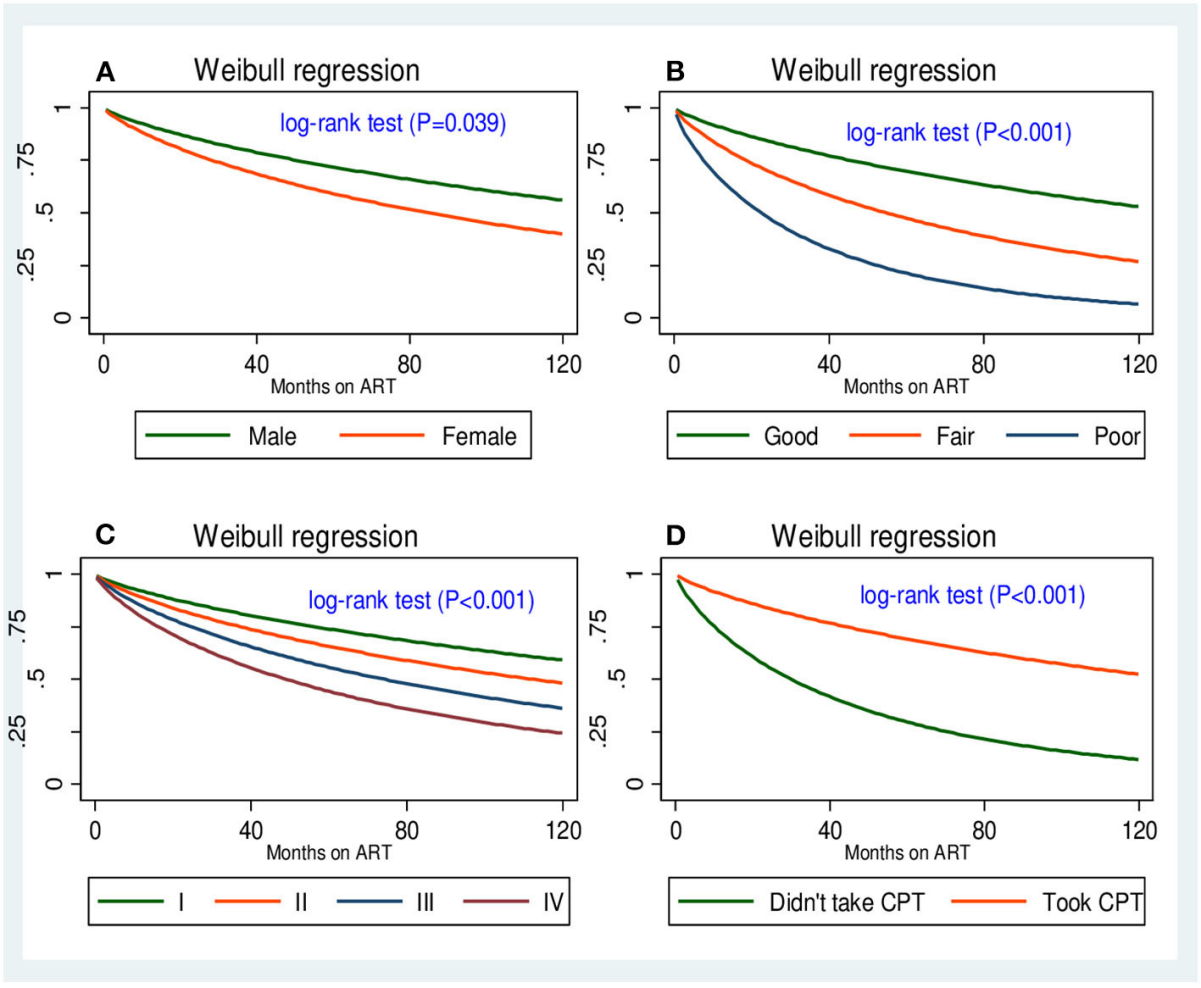
Four survival functions corresponding to **(A)** gender, **(B)** adherence to ART, **(C)** WHO HIV clinical stage, and **(D)** CPT.

The Kaplan–Meier estimation method was used to determine the overall survival time. As such, the median OI-free survival time after the initiation of ART was ~95.8 months (7.87 years) (Figure 3). The survival probability past 6, 12, and 24 months was 93.74% (95% CI: 91.27, 95.53%), 87.74% (95% CI: 84.51, 90.33%), and 79.01% (95% CI: 79.73, 86.44%), respectively. Furthermore, tests for survival function equality were conducted to compare the OI-free survival probability at each point in time between categories of covariates of OIs predictor variables, and the existence of a difference was determined using the Mantel–Cox (log-rank) test at *p* < 0.05. Thus, there was a statistically significant difference in developing OIs across the gender, adherence to ART, WHO HIV clinical stages, and history of taking CPT (Figures 4A–D).

### Predictors of opportunistic infections

The multivariable Weibull regression model revealed that being female, not taking CPT, poor ART adherence, mild malnutrition, and WHO HIV clinical stage III turned out to be independent predictors of OI acquisition. To begin with, compared to male subjects, female subjects had a 65% [AHR: 1.65 (95% CI (1.15, 2.36), *P* = 0.007)] higher hazard of developing OIs. In addition, the hazards of developing OIs were 98% higher in adolescent and adult PLHIV with WHO HIV clinical stage III defining illnesses than in adult PLHIV with WHO HIV clinical stage I [AHR: 1.98 (95% CI (1.12, 3.51), *P* = 0.019)]. In this study, we also found that the hazards of OIs occurring at any given time were three times higher in PLHIV who had not initiated CPT than in their counterparts [AHR: 2.58 (95% CI (1.62, 4.11), *P* < 0.001)].

Furthermore, a correlation between malnutrition and OIs was identified. Adolescents and adults with mild malnutrition had a 62 % higher hazard of developing OIs [AHR: 1.62 (95% CI (1.06, 2.54), *P* = 0.035)] than PLHIV with no malnutrition. The level of ART adherence was also found to be an independent predictor of OIs in the multivariable analysis. Thus, the hazards of developing OIs were four times higher in adolescents and adults with poor ART adherence compared to those with good ART adherence [AHR: 4.21 (95% CI (2.39, 7.44), *P* < 0.001); Table 3].

**Table 3:** Bivariable and multivariable Weibull regression analyses of OI predictors among adolescents and adults after the initiation of ART at WSUCSH, Southern Ethiopia, 2012–2021 (*n* = 515).

**Characteristics**	**Status**		**Bivariable analysis**	**Multivariable analysis**	
	**Censored *n* (%)**	**Event *n* (%)**	**CHR (95% CI)**	**AHR (95% CI)**	***P-*value**
**Age (years)**					
15–24	39 (61.9)	24 (38.1)	1	1	1
25–34	178 (72.4)	68 (27.6)	0.65 (0.41, 1.03)	0.75 (0.45, 1.26)	0.277
35–44	90 (60.0)	60 (40.0)	1.00 (0.62, 1.61)	1.15 (0.68, 1.97)	0.592
45–54	36 (83.7)	7 (16.3)	0.40 (0.17, 0.93)	0.77 (0.31, 1.89)	0.564
≥55	8 (61.5)	5 (38.5)	0.78 (0.30, 2.05)	2.02 (0.71, 5.78)	0.188
**Educational level**					
No education	72 (67.9)	34 (32.1)	1.44 (0.79, 2.61)	0.94 (0.50, 1.78)	0.861
Primary	129 (64.2)	72 (35.84)	2.03 (1.18, 3.50)	1.90 (0.60, 1.99)	0.778
Secondary	84 (66.7)	42 (33.3)	1.66 (0.93, 2.95)	1.44 (0.78, 2.64)	0.245
Tertiary and above	66 (80.5)	16 (19.5)	1	1	1
**Gender**					
Male	163 (74.1)	57 (25.9)	1	1	1
Female	188 (63.7)	107 (36.3)	1.59 (1.150, 2.19)	1.65 (1.15, 2.36)	0.007*
**Isoniazid preventive therapy**				
No	87 (48.1)	94 (51.9)	2.26 (1.66, 3.08)	1.21 (0.83, 1.76)	0.312
Yes	264 (79.0)	70 (21.0)	1		
**Cotrimoxazole preventive therapy**					
No	13 (26.5)	36 (73.4)	3.30 (2.27, 4.77)	2.58 (1.62, 4.11)	< 0.001*
Yes	338 (72.5)	128 (27.4)	1	1	1
**Nutritional status**					
Normal	218 (71.2)	88 (28.8)	1		
Mild malnutrition	52 (65.0)	28 (35.0)	1.61 (1.05, 2.47)	1.62 (1.06, 2.54)	0.035*
Moderate malnutrition	18 (54.6)	15 (45.5)	2.09 (1.21, 3.62)	1.45 (0.79, 2.67)	0.228
Severe malnutrition	24 (52.2)	22 (47.8)	2.52 (1.58, 4.03)	1.30 (0.75, 2.25)	0.353
Overweight	33 (78.6)	9 (21.4)	0.70 (0.35, 1.40)	0.63 (0.31, 1.29)	0.206
Obese	6 (75)	2 (25)	1.20 (0.30, 4.88)	1.43 (0.34, 6.08)	0.627
**WHO HIV clinical stage**					
Stage I	186 (82.3)	40 (17.7)	1	1	1
Stage II	66 (60.6)	43 (39.5)	1.91 (1.24, 2.95)	1.21 (0.68, 2.15)	0.528
Stage III	75 (49.3)	77 (50.7)	3.43 (2.34, 5.02)	1.98 (1.12, 3.51)	0.019*
Stage IV	24 (85.2)	4 (14.3)	0.66 (0.24, 1.86)	0.41 (0.12, 1.43)	0.161
**ART adherence**					
Good	332 (73.8)	118 (26.2)	1	1	1
Fair	13 (33.3)	26 (66.7)	2.24 (1.46, 3.42)	1.45 (0.86, 2.46)	0.163
Poor	6 (23.1)	20 (76.9)	4.14 (2.57, 6.67)	4.21 (2.39, 7.44)	< 0.001*
**Baseline ART regimen**					
AZT + 3TC + NVP	12 (41.4)	17 (58.6)	1.55 (0.77, 3.11)	1.08 (0.49, 2.37)	0.842
AZT + 3TC + EFV	5 (33.3)	10 (66.7)	2.46 (1.11, 5.46)	1.05 (0.39, 2.81)	0.927
TDF + 3TC + EFV	219 (66.4)	111 (33.6)	1.06 (0.62, 1.81)	0.95 (0.53, 1.71)	0.876
TDF + 3TC + NVP	11 (53.4)	10 (47.6)	1.99 (0.90, 4.41)	0.70 (0.28, 1.74)	0.445
TDF + 3TC + DTG	104 (86.7)	16 (13.3)	1	1	1
**Comorbidity**					
No	344 (68.8)	156 (31.2)	1	1	1
Yes	7 (46.7)	8 (53.3)	1.63 (0.80, 3.31)	1.06 (0.49, 2.32)	0.884
**Functional status**					
Working	302 (73.1)	111 (26.9)	1	1	1
Ambulatory	40 (43.5)	52 (56.5)	2.22 (1.59, 3.08)	0.87 (0.55, 1.37)	0.535
Bedridden	9 (90.0)	1 (10.0)	0.37 (0.52, 2.66)	0.62 (0.69, 5.63)	0.674
**Presence of OIs at enrollment**					
Yes	173 (57.1)	34 (16.0)	2.53 (1.74, 3.69)	1.62 (0.94, 2.80)	0.083
No	178 (84.0)	130 (42.9)	1	1	1

Reference category-1; ^*^statistically significant at P < 0.05.

## Discussion

This retrospective cohort study sought to determine the incidences of OIs and identify potential predictors among adolescents and adults after the initiation of ART at Wolaita Sodo University Comprehensive Specialized Hospital. During the 10 years of follow-up periods, the overall incidence rate of OIs after ART initiation was 8.97 cases (95% CI: 7.69, 10.44) per 100 PYO.

The rate of OIs in our study was higher than that found in other cohort studies' findings from the northern parts of Ethiopia, the Tigray region, Mekelle (7.5 cases per 100 PYO) (32); Arba Minch town, South Ethiopia (3.4 cases per 100 PYO) (28); Uganda (5.9 cases per 100 PYO) (25); a collaborative observational cohort study involving 15 sites in the Asia and Pacific region (7.3 cases per 100 PYO) (34); multicohort analysis of persons infected with HIV in the United States and Canada, 2000–2010 (2.3 cases per 100 PYO) (21).

The observed difference in the rates of OIs across these studies could be due to sample size and a short follow-up period in some studies. In the Mekelle study, for instance, the cohort was followed for 36 months only. Another possible explanation could be due to a disparity in the level of engagement in HIV care in low-income countries and western countries. Furthermore, the incidence of OIs in the current study was also higher than study findings in Poland, with 6.8, 6.5, and 4.8 cases per 100 PYO in 2000, 2001, and 2002, respectively (19). In contrast, the current study's findings are lower than previous studies in Dessie, north Ethiopia (13.5 cases per 100 PYO) (27), Taiwan (17.88 cases per 100 PYO) (39), and Pune, India (35.7 cases per 100 PYO) (40). The potential explanation is that the majority of PLHIV at ART enrollment were younger age groups, where individuals infected with HIV at a younger age may remain free of clinical AIDS for more than 20 years.

In this study, community-acquired pneumonia (41.5%) was the most common OI, followed by pulmonary tuberculosis (22.2%). In contrast, pulmonary tuberculosis was the most common OI compared to other studies conducted in Ethiopia including St. Hospital, Paul's Millennium Medical College, Addis Ababa (43.49%) (41), and Ayder Referral Hospital, Mekelle (32.3%) (32). The fact that the incidence of tuberculosis was lower just after community-acquired pneumonia may be attributable to the intensified tuberculosis screening program and the provision of IPT, which was more than 80% in this study. Furthermore, about 73% of patients who were not initiated on CPT exhibited OI, which might have contributed to the higher proportion of pneumococcal infection.

In this analysis, we found that participants who were not initiated on CPT, poor ART adherence, WHO HIV clinical stage III, mild malnutrition, and female PLHIV were independent predictors of OIs after ART commencement. To begin with, the hazards of developing OIs were 4-fold greater in PLHIV with poor ART adherence compared to those who had good ART adherence. This finding was congruent with previous research findings in Ethiopia (27, 32) and Ilorin, Nigeria (42). This is because poor adherence to ART reduces the effectiveness of ARV drugs while also accelerating viral replication and immune suppression, creating a favorable environment for the development of OIs. Suppression of HIV replication is an important component in prolonging and improving the quality of life for the patient and minimizing the risk of transmission of HIV to others. Adequate suppression of HIV replication requires strict adherence to prescribed regimens of antiretroviral drugs.

Furthermore, WHO HIV clinical stage at presentation was an independent predictor of OIs. The hazards of developing OIs were 98% higher in PLHIV who presented for medical treatment at a chronic HIV care and treatment center with advanced WHO clinical stage of the disease. This finding is consistent with a study in Dessie Comprehensive Specialized Hospital, North Ethiopia (27). This can be explained by the fact that the progression of HIV from infection through subtle symptoms to advanced HIV disease is correlated with prolonged immune suppression, which is partly a reflection of late presentation to health facilities. In addition, individuals with advanced HIV diseases are prone to acquiring multiple other OIs.

Our study also identified that adult PLHIV who did not take CPT had three times higher hazards of developing OIs compared to their counterparts. Compelling evidence supporting the current finding was reported in other studies (43–46). In addition, a retrospective cohort study by Bizuayehu et al. revealed that the hazards of OIs were reduced by 69% in adult patients with HIV who took CPT (44). The Ethiopian ART guideline recommends initiating trimethoprim–sulfamethoxazole as the primary prevention of PCP, toxoplasmosis, bacterial infections, malaria, and diarrhea caused by Isospora belli or Cyclospora species. This recommendation was made as part of a package of HIV-related services (10). Moreover, on top of the appropriate use of potent ART, the prophylactic use of cotrimoxazole is of critical importance in providing each patient with the best opportunity to live a long and healthy life with HIV infection. Nevertheless, the reverse is valid if patients infected with HIV did not receive CPT.

Malnutrition was also identified as an independent predictor of OI acquisition in this cohort study. Adolescents and adults with mild malnutrition had 62% higher adjusted hazards of developing OIs compared to those who had normal BMI, holding other factors constant. This finding was in agreement with previous studies in KwaZulu-Natal, South Africa, where low BMI was associated with morbidity and mortality among patients with HIV (47). This could be due to a complex and mutually reinforcing relationship between malnutrition and HIV infection. Furthermore, malnutrition can result from HIV-induced immune impairment, and the resulting OIs can cause poor appetite and nutrition absorption from the gastrointestinal.

Finally, statistical analysis of this result revealed a positive relationship between gender and OIs. Female PLHIV had a 65% higher hazard of developing OIs than male PLHIV. This finding was in contrast to a cohort study in Arba Minch, Southern Ethiopia, where the adjusted hazard of OI acquisition was two times greater in men (28). Moreover, in our study, a higher proportion of female PLHIV (53.85%) was poorly adherent to prescribed ART drugs, and studies in Ethiopia (48) and Nigeria (49, 50) reported that being female was associated with non-adherence to ART compared to men, which may partly explain the correlation of being female with the occurrence of OIs.

### The strength and limitations of this study

This study provides important information regarding the role of clinical, laboratory, and treatment-related variables in the acquisition of OIs. It could identify the lacunae in the existing data and missing data points because of the use of data collection software. Furthermore, the proportion of losses to follow-up was low (< 3%). No study addressed current issues in the study setting. In addition, the study design was efficient for ascertaining temporal relations. We also acknowledged that the current study has some limitations that should be interpreted with caution. First, as is common in resource-limited settings, some of the OIs were diagnosed clinically only, which may overestimate or underestimate some morbidity. Second, due to the study's retrospective nature, we were unable to investigate the behavioral and environmental-related independent factors that may influence OI acquisition. Third, because of the “test and treat” policy implemented following 2016 WHO recommendation, the baseline CD4+ T lymphocyte count was not recorded for a total of 187 (36.3%) patients, resulting in non-random missing data.

## Conclusion and recommendations

We found that the rate of OIs after the initiation of ART was still high. Moreover, poor adherence to ART, mild malnutrition, advanced HIV disease at presentation, being female, and not taking CPT were independent predictors of OIs. We recommend that health workers caring for PLHIV assess and provide intensified care for patients presenting with advanced HIV disease and malnutrition to avoid the risk of developing OIs after ART initiation. Furthermore, enhanced adherence counseling should be provided by the ART team based on the patient's adherence track records. Prescription of CPT for patients with ART should be instituted as indicated in the guidelines for the ART program and should be readily accessible to increase survival among patients with HIV. The Wolaita zone health department should strengthen public awareness to foster early presentation to health facilities before advanced HIV disease supervenes, which in turn curbs the frequent development of OIs that follow marked immune compromise at HIV diagnosis. Moreover, national HIV program mainstreaming managers and policymakers should intensify comprehensive HIV care and support activities. Finally, prospective observational studies are required to assess the role of personal, social, or environmental factors in the occurrence of OIs.

## Data availability statement

The original contributions presented in the study are included in the article/supplementary material, further inquiries can be directed to the corresponding author.

## Ethics statement

The studies involving human participants were reviewed and approved by Institutional Review Board of Wolaita Sodo University. Written informed consent for participation was not required for this study in accordance with the national legislation and the institutional requirements.

## Author contributions

BW: conceptualization, project administration, software, formal analysis, visualization, and writing—original draft. BW, CD, BY, and YA: data curation and validation. BW, CD, BY, AK, and YA: methodology and writing—reviewing and editing. AK: supervision. All authors contributed to the article and approved the submitted version.
